# Immune conversations at the border: meningeal immunity in health and disease

**DOI:** 10.3389/fimmu.2025.1531068

**Published:** 2025-01-29

**Authors:** Preya U. Patel, Aryan Regmi, Angelina I. Dass, Olga L. Rojas

**Affiliations:** ^1^ Department of Immunology, University of Toronto, Toronto, ON, Canada; ^2^ Krembil Research Institute, University Health Network, Toronto, ON, Canada

**Keywords:** meningeal anatomy, meningeal immunity, central nervous system, immune cells - dura mater, CNS diseases

## Abstract

The brain and spinal cord, collectively known as the central nervous system, are encapsulated by an overlapping series of membranes known as the meninges. Once considered primarily a physical barrier for central nervous system protection, the bordering meninges are now recognized as highly immunologically active. The meninges host diverse resident immune cells and serve as a critical interface with peripheral immunity, playing multifaceted roles in maintaining central nervous system homeostasis, responding to pathogenic threats, and neurological disorders. This review summarizes recent advancements in our understanding of meningeal immunity including its structural composition, physiological functions, and role in health and disease.

## 1 Introduction

The central nervous system (CNS), formed by the brain and spinal cord, is a highly specialized and sensitive system protected by the blood-brain barrier (BBB), a semipermeable microvessel system which restricts molecular, microbial, and immunological access to the CNS from peripheral circulation. Growing evidence in the field shows how the BBB is not the only interface involved as a barrier system in the CNS. The CNS is encapsulated by a three-layered membrane system called the meninges and further protected by the vertebrae or the skull (1). In addition to providing structural support, it has become abundantly clear that the meninges are areas with high immunological activity. Meningeal immunity encompasses a complex and dynamic system that includes a wide repertoire of immune cells, lymphatics, permeable vasculature, and cytokine signaling pathways. Under steady-state conditions, meningeal lymphoid and myeloid populations provide immune surveillance and primary defense against pathogens, while also supporting cognitive and behavioral functions (2–5). Advanced imaging techniques and single-cell sequencing technologies have offered unprecedented insight into the specialized architecture and immune landscape of the meninges. It was initially thought that meningeal lymphocytes were exclusively derived from the periphery, but recent evidence has shown that the bone marrow of the skull calvaria is an important source of meningeal immune cells (6–8). Furthermore, organized lymphoid structures within the meninges at steady-state that may support the development of immune cells and facilitate local antigen-presentation in the meninges have been recently identified (9). Differences in the meningeal immune milieu have been described with aging as well as with neurological diseases like multiple sclerosis, viral infections, and neurodegenerative diseases, further suggesting the meninges are a unique immune niche within the CNS. This review provides an updated understanding of meningeal immunity, covering its roles in health and disease. A summary of key anatomical features of the meningeal compartment is first provided, followed by a detailed description of the immune cell subsets involved in the meninges at homeostasis, and emerging evidence about their spatial organization. We further discuss changes in meningeal immunity during various neurological conditions, concluding with research gaps and future directions.

## 2 Anatomy of the meninges and contribution to mobilization of immune cells

**Figure 1:**
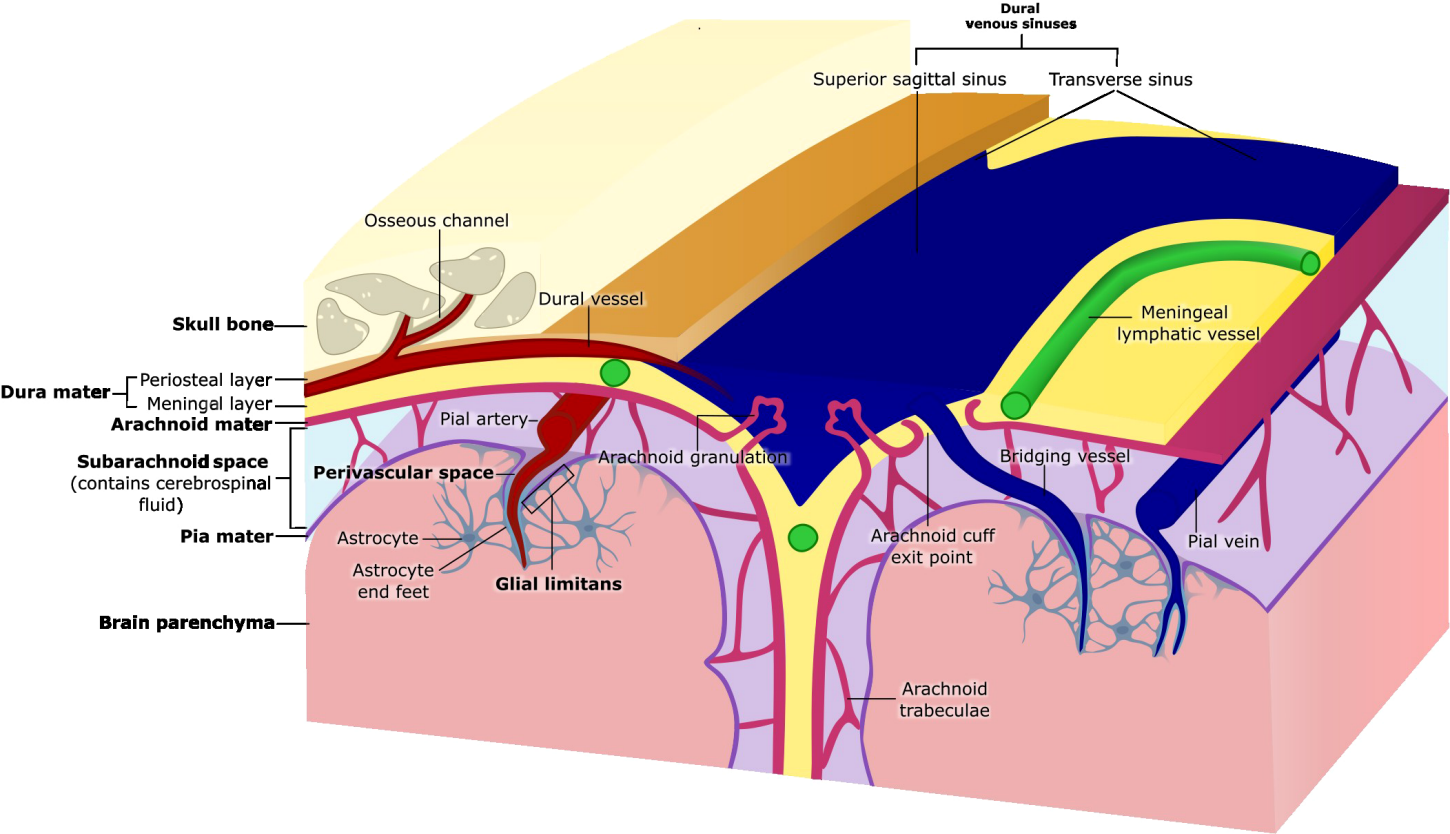
Anatomy of the meninges. The meninges are three membranous layers that cover the brain and spinal cord. The dura mater, the outermost layer, consists of a periosteal layer and a meningeal layer, which separate to form the dural venous sinuses (e.g., superior sagittal and transverse sinuses). Beneath the dura lies the arachnoid mater, a thin tissue layer which forms the arachnoid granulations that protrude into the dura mater, and arachnoid trabeculae that traverse the subarachnoid space and attach to the pia mater. The subarachnoid space contains cerebrospinal fluid (CSF) and is crossed by bridging vessels that supply the brain as well as drain blood from the brain into the dural venous sinuses. Arachnoid cuff exit points are openings that form where bridging vessels cross the arachnoid mater into the subarachnoid space. The pia mater, the innermost meningeal layer, adheres closely to the surface of the brain. The glial limitans is a thin layer of astrocytic end feet connected by tight junctions that lies directly beneath the pia mater. The perivascular space surrounding pial blood vessels is also lined by pia mater (coverage not shown), that then becomes continuous with the glial limitans as the vessels penetrate the brain parenchyma.

The meninges are composed of three structurally distinct layers: the dura mater, arachnoid mater and pia mater (**Figure 1**). Together, these layers were thought to exist to provide structural support to the brain; however, a growing body of evidence supports the idea that the meninges play critical roles in immune surveillance and homeostatic functions of the CNS.

The outermost layer of the meninges, the dura mater, is a dense, collagenous, and highly vascularized membrane. The dura mater is composed of the periosteal layer that lies adjacent to the skull, and the meningeal layer. The periosteal layer contains fibroblasts and osteoblasts which secrete copious amounts of collagen, providing the dura with strength (10). The two layers of the dura are mostly fused, but they separate to give way to large dural sinuses including the superior sagittal, transverse, and occipital sinuses that are essential to drain blood and cerebrospinal fluid (CSF) from the brain back into systemic circulation (1). Intracranial bridging veins that drain blood from the brain also empty into the dural venous sinuses (1) (**Figure 1**). The blood supply of the dura derives mainly from the meningeal arteries in both humans and mice, and the dural vasculature is connected to the skull bone marrow through bone encased vascular channels, referred to as osseous channels (6, 7, 11). The blood vessels in the dura are unique compared to the other vessels in the CNS, as they are fenestrated and lack tight junctions, permitting the movement of relatively large molecules from the blood into the dura mater (12). Furthermore, recent work has characterized an extensive *bona fide* dural lymphatic vessel network in the dorsal and basal part of the skull that drain CSF and meningeal immune cells into the deep cervical lymph nodes (dCLNs), and partly the superficial cervical lymph nodes (13–15). Dural lymphatic vessels, also referred to as meningeal lymphatic vessels (MLV), in mice are characterized by the expression of classical endothelial markers, including Prospero homeobox protein 1 (Prox1), lymphatic vessel endothelial hyaluronan receptor 1 (Lyve1), Podoplanin (gp38), and vascular endothelial growth factor receptor (VEGFR) (14, 15). Interestingly, dorsal and basal meningeal lymphatic vessels (MLVs) differ morphologically. Basal MLVs have blunt-ended capillaries and lymphatic valves–features that, along with their position near the underlying subarachnoid mater, make them better suited for CSF uptake and drainage than dorsal MLVs (14). Similar structures have been identified in humans through the use of magnetic resonance imaging (16). While a deeper characterization of drainage pathways from the meninges to the cervical lymph nodes in humans is still required, it appears that in contrast to mice, CSF drainage in humans occurs primarily through the dorsal MLVs near the dural sinuses (15–17). The efflux of CSF to the dura mater is not only important for the drainage of waste, but also for the presentation of brain-derived antigens to dural immune cells and communication with the adjacent skull bone marrow (4, 13–15, 18).

Beneath the dura mater lies the arachnoid mater, a thin fibrous membrane named for its spider web-like appearance (19). This membrane consists of layers of flattened epithelial cells bound by tight junctions and is separated from the underlying pia mater by the subarachnoid space (SAS) (20) (**Figure 1**). The SAS is filled with CSF that provides buoyancy and support to the brain by regulating cerebral blood flow and facilitating the drainage of waste (14, 21–23). CSF in the SAS can be drained to the cervical lymph nodes by meningeal lymphatics located in the dural layer or may enter the dural sinuses through arachnoid granulations (24). Fibroblast-like cells and collagenous projections produced by cells of the arachnoid mater called arachnoid trabeculae, anchor the arachnoid mater to the pia mater, allowing CSF to circulate within the SAS (25) (**Figure 1**). By using transgenic mice to delineate arachnoid barrier cells, microscopy techniques, and fluorescent tracers to track CSF movement, researchers recently demonstrated gaps in the arachnoid membrane around cerebral bridging veins. These gaps, termed arachnoid cuff exit (ACE) points, may enable the bulk movement of CSF and molecules directly between the dura and the SAS (26) (**Figure 1**).

The innermost meningeal layer, the pia mater, is a delicate membrane composed of endothelial and fibroblast-like cells that adheres tightly to the surface of the brain and spinal cord (1, 27). Arteries in the SAS extend through the pia mater, forming arterioles that can penetrate the brain parenchyma (28) (**Figure 1**). These blood vessels are surrounded by fluid-filled space, known as the perivascular space or Virchow-Robin space, acting as conduits of the glymphatic system which is postulated to facilitate exchange of CSF and brain interstitial fluid and enable waste clearance including at capillary level (27, 29, 30). The pia envelopes most of the blood vessels traversing through the SAS, and the lack of tight junctions within the pia mater allows for the movement of molecules and fluid between the pia, perivascular space and the SAS, enabling communication between all three compartments (29, 31). Astrocyte projections line the underside of the pial membrane, forming another CNS barrier known as the glial limitans (32) (**Figure 1**). Similarly, these astrocytes end-feet wrap all parenchyma-permeating blood vessels. The function of this barrier is to further regulate the movement of molecules from the CSF into the CNS parenchyma (29, 32, 33). Together, the pia mater and the arachnoid mater form the leptomeninges, which are considerably thinner than the dura and cover the brain and spinal cord’s surface (1, 34).

## 3 Immune composition of the meninges at steady-state

**Figure 2:**
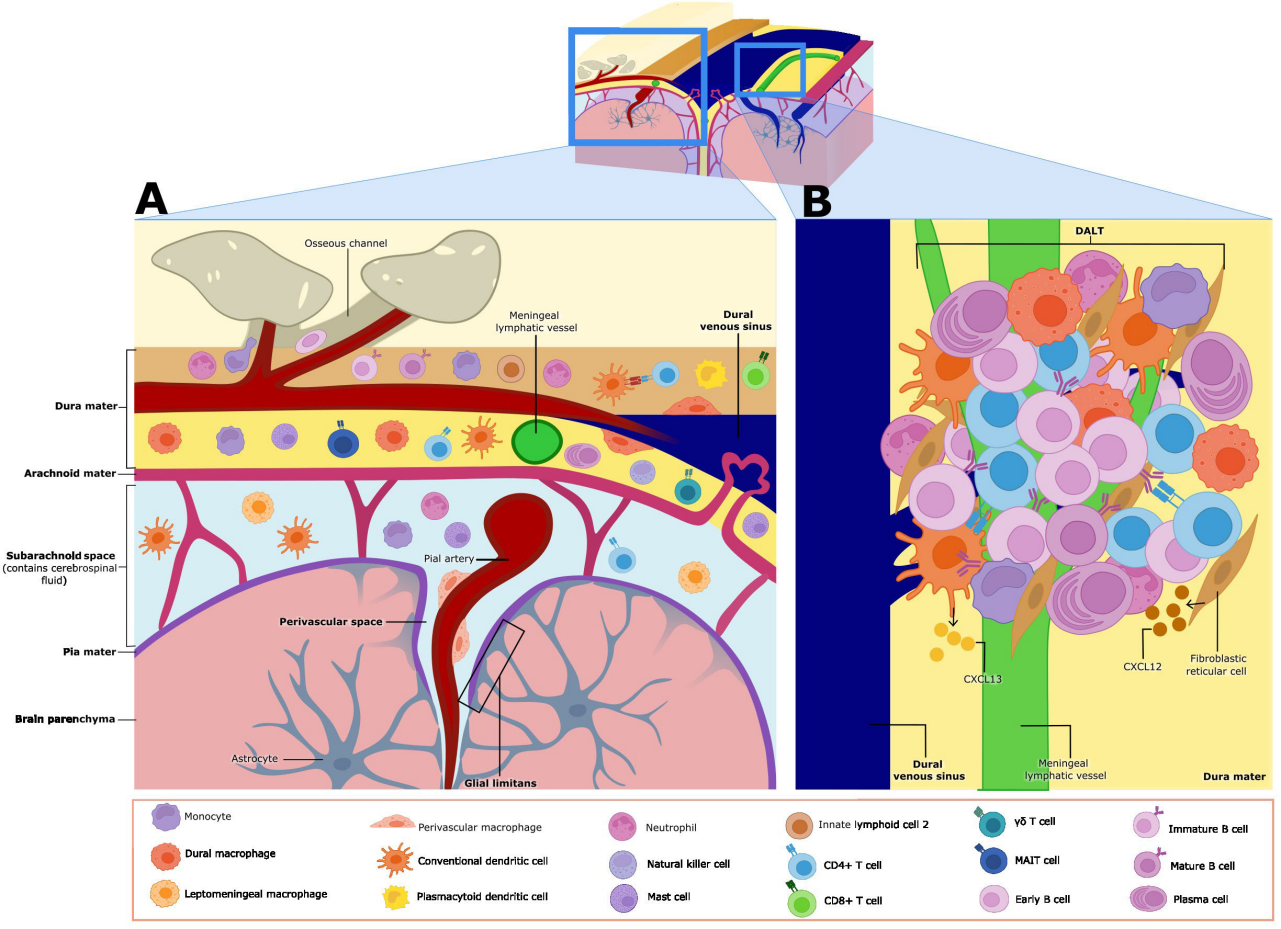
Meningeal immune cell niche at homeostasis. **(A)** The meninges contain various subsets of immune cells during homeostasis. γδ, CD8, CD4, and MAIT T cells are found in the dura mater and the leptomeninges (made up of the arachnoid membrane, subarachnoid space, and the pia mater–leptomeningeal cells are shown only in the subarachnoid space in this figure). Monocytes, conventional dendritic cells (cDCs), natural killer (NK) cells and type 2 innate lymphoid cells (ILC2s) are also found in both the dura mater and leptomeninges. Neutrophils, plasma cells, plasmacytoid dendritic cells (pDCs), and dural macrophages (dmMφ) have only been described in the dura mater. Leptomeningeal macrophages (mMφ) and perivascular macrophages are found in leptomeninges and the perivascular Virchow-Robin space respectively. Mature B cells are found in both regions, but immature and early B cells have only been described in the dura mater. Meningeal cells play key roles in immune surveillance and some subsets influence brain function and behavior through cytokine secretion. **(B)** Closer view of dural-associated lymphoid tissue (DALT). DALTs are immune cell aggregates found along the dural venous sinuses, and they contain activated B cells and plasma cells. DALTs also contain other immune subsets, such as conventional dendritic cells (cDCs), monocytes, neutrophils, and CD4^+^ T cells. Stromal cells like fibroblastic reticular cells and follicular dendritic cells, provide structural support to DALT, as well as release chemoattractants such as CXCL13 and potentially CXCL12, which may contribute to the formation, activation and expansion of these structures. DALTs are strategically positioned around the dural venous sinuses and ACE points, likely to mount rapid and robust immune responses against pathogens.

Due to its distinctive fenestrated vasculature and lymphatic system, the dura mater harbors a substantial population of immune cells within the meninges during homeostatic conditions (**Figure 2A**). In wild-type young adult mice at steady-state, dural CD45+ immune cells isolated by mechanical dissociation consist of neutrophils (43.7%), B cells (26.3%), T cells (3.2% double negative, 2.8% CD8^+^, and 1.7% CD4^+^), monocytes (2.5% Ly6C^+^, 1.2% Ly6C^−^), natural killer cells (1.6%), macrophages (1.3%), mast cells (1.1%), plasmacytoid dendritic cells (pDCs) (1%), classical dendritic cells (cDCs) (0.9%), type 2 innate lymphoid cells (ILC2) (0.4%), and plasma cells (0.3%) (7) (**Figure 2A**). It is important to highlight that enzymatic tissue dissociation of the dura has been associated with small differences in the immune cell composition, including an increased frequency of neutrophils, mast cells, and DCs (35). Meningeal immune cells are distributed throughout the meningeal layers, with a notable tendency to localize around the dural venous sinuses under steady-state conditions (4, 5, 7, 9, 35). Recent research has demonstrated that some immune cells form organized clusters along the dural sinuses, known as dural-associated lymphoid tissues (DALT) (4, 5, 9) (**Figure 2B**). In contrast to the dura, the leptomeninges feature tight junctions and low expression of adherence molecules under homeostasis to tightly regulate leukocyte trafficking (1, 34). The leptomeninges hosts unique macrophage and dendritic cell (DC) subsets, as well as a few lymphoid cells, but considerably less B cells than the dura (36, 37) (**Figure 2A**). While leptomeninges are implicated in neurological diseases such as meningitis and MS, their role in meningeal immunity under homeostatic conditions remains poorly understood (38, 39). Below we highlight what is known in the literature about the most prominent adaptive and innate immune cells in the meningeal compartment under steady-state conditions, and our understanding of the spatial organization of select meningeal lymphocyte populations.

### 3.1 T cells

While T cells are mostly absent from the brain parenchyma, they are readily detected in the murine meninges during steady state (4). Whole-mount immunohistochemistry and two-photon imaging in a live mouse revealed that T cells are not evenly distributed throughout the dural tissue but are highly localized to regions around the dural sinuses (4). Parabiotic studies have indicated that CD4^+^ and CD8^+^ T cells are continuously replaced in the homeostatic dural meninges from peripheral blood (4, 40). There was preferential infiltration of circulating T cells from the dural sinuses, and to a lesser extent along cerebral veins projecting into the sinus (4). Endothelial cells forming the dural sinus vasculature expressed lower amounts of tight junction proteins and high amounts of integrins which favored T cell adherence and extravasation (4). The dural stromal composition—including endothelial cells, mural cells, fibroblast-like cells, and stroma-derived chemokines—further promoted homeostatic T cell recruitment and retention (4). For example, *Cxcl12* and *Cxcl16* were highly expressed in the meninges and involved in the recruitment of circulating CXCR4 and CXCR6-expressing leukocytes (4). Following resection of the dCLNs, wild-type mice had substantially greater accumulation of CD4^+^ T cells in the meninges, suggesting that draining lymph nodes represent a critical exit point for these cells (40). Dural CD4^+^ T cells are polarized towards Th1, Th2, Th17 and regulatory T (T_reg_) cell subsets, which suggests that a balance between pro- and anti-inflammatory subsets exists at homeostasis (**Table 1**) (4).

**Table 1:** Key immune cell subsets in the meninges of central nervous system during homeostasis.

Immune Cell subset	Location	Origin	Role	References
T cells (CD4, CD8, MAIT cell, γδ T cell)	Dura (4-7.7%) Leptomeninges (3%)	Peripheral circulation	Cognition and behavior Immune surveillance	(33–39) (4, 51, 171)
B cells and plasma cells	Dura (7-27%) Leptomeninges (1%)	Skull bone marrow Peripheral circulation	Immune surveillance and dural-associated lymphoid tissue structure Regulate stress-like behavior	(5, 7, 8, 51, 52) (45)
Leptomeningeal Macrophages (mMφ)	Leptomeninges	Embryonic Yolk Sac (development) Self-Renewal (adulthood)	Immune surveillance Monitoring and filtering the CSF	(46) (68)
Dural Macrophages (dmMφ)	Dura	Embryonic Yolk Sac (development) Blood Monocytes (adulthood)	Immune surveillance	(46)
Perivascular Macrophages (pvMφ)	Virchow–Robin Spaces	Postnatal seeding by mMφ (development) Self-Renewal (adulthood)	Immune surveillance Monitoring and filtering the CSF Regulate endothelial permeability? Regulation of systemic immunity Regulation of lipid and glucose metabolism within the CNS	(46) (68–71) (72, 73) (74–76) (77, 78)
Dendritic Cells (DCs)	Dura (1.9-19%) Leptomeninges (2.6%)	Skull bone marrow Peripheral circulation	Antigen presentation to T cells	(4, 7, 51, 78)
Neutrophils	Dura (12-43.7%)	Skull bone marrow Peripheral circulation	Immune surveillance Production of ROS, enzymes and chemoattractants	(6, 7, 51, 78) (81)
Mast Cells	Dura (1.1-2%)	Skull bone marrow Peripheral circulation	Cognition and glial cell function	(7, 51, 84, 85)
Innate lymphoid cells (ILCs)	Dura (0.4%)	Skull bone marrow Peripheral circulation	Motor and psychiatric function	(7, 86, 87)
Natural Killer Cells (NKs)	Dura (1.6-4%) Leptomeninges (1%)	Skull bone marrow Peripheral circulation	IFNγ expression to astrocytes	(7, 51, 86, 93, 94)

Several subsets of meningeal T cells have been shown to play critical roles in cognitive function and neurogenic development, likely representing an evolutionary link between the nervous and immune systems. The Morris Water Maze (MWM) test is widely used in neuroscience research to evaluate spatial learning and memory in mice. In wild-type mice, the performance of the MWM test led to the accumulation of meningeal interleukin (IL)-4-producing CD4^+^ T cells (2). Pharmacological depletion of meningeal T cells or using IL-4 deficient mice resulted in learning deficits that could be reversed with intraperitoneal injection of wild-type, but not IL-4-deficient, CD4^+^ T cells (2). Meningeal T cell release of IL-4 may support learning by promoting hippocampal brain-derived neurotrophic factor (BDNF) release and neurogenesis (41). Specifically, this effect may be mediated by astrocytes which border the meninges and can respond to meningeal cytokine release. Indeed, IL-4-treated astrocytes *in vitro* produced more BDNF and hippocampi from mice tested using memory and cognition tasks had increased BDNF mRNA, which was absent in IL-4-deficient mice (2, 41). However, the direct application of IL-4 inhibited adult hippocampal neural stem cell proliferation *in vitro* (42). Work by Radjavi A et al. suggested that T cell antigen specificity may be required to support learning and cognition (40). Mice with a limited CD4^+^ T cell repertoire restricted to reactivity against chicken egg ovalbumin antigen (OTII mice) had impaired hippocampal neurogenesis and memory despite elevated CNS IL-4 levels (42, 43). However, the transfer of T cells reactive to a common CNS antigen into OTII mice improved performance in the MWM test (43). In support, resection of the dCLNs, which likely limited brain-derived antigen sampling, impaired learning and memory function in wild-type mice subjected to the MWM test despite a greater accumulation of CD4^+^ T cells in the meninges (40). Together, these studies indicate that homeostatic levels of CD4^+^ T cell cytokines, including IL-4, that is lost in mice with limited T cell repertoires may be required to support hippocampal-based memory and learning functions. While CD4^+^ T cells directed against CNS-derived self-antigen may be sufficient to rescue MWM task performance at least in animals with clonally restricted T cell repertoires, this does not preclude the requirement of other antigenic specificities of CD4^+^ T cell populations, and the nuances of T cell specificity to support cognitive functions remains largely unknown (40).

Furthermore, interferon-gamma (IFN-γ) production by meningeal T cells has been linked to regulating social behavior in mice (44). There is a high frequency of T cells in the healthy meninges that produce IFN-γ. Infiltrating meningeal T cells may use the integrin VLA-4 to cross vasculature and enter the meningeal space (44). The administration of anti-VLA-4 antibody partially depleted meningeal T cells during steady-state, and this was sufficient to impair social behavior in mice measured using the three-chamber sociability test. Mice that were IFN-γ- deficient (IFN-γ^−/−^) exhibited similar social deficits, further highlighting that T cell-derived IFN-γ supports social behavior (44). Social interactions are required for the survival of a species, but they also increase the likelihood of pathogen transmission. Therefore, it is plausible that this aspect of pathogen defense in the meninges, mediated by IFN-γ, may have coevolved with social behavior to provide mutual benefits (44).

Additionally, IL-17a-secreting meningeal γδ (γδ17) T cells, have been implicated in controlling short-term memory and anxiety-like behavior during steady-state in mice (45, 46). Located along the dural sinuses, γδ17 T cells are reported to produce about 90% of IL-17a that is found within the meninges during steady-state conditions (46). These cells originate in the fetal-thymus and populate the meninges shortly after birth in a CXCR6-dependent manner (45, 46). Notably, these cells are self-renewing and not replenished by contributions from the periphery (46). The production of IL-17a by meningeal γδ17 T cells was first implicated in the promotion of short-term memory through increasing glutamatergic synaptic plasticity of the hippocampal neurons (45). Short-term memory, as assessed by the Y maze, was impaired in full-body IL-17a or γδ-deficient mice, bone marrow chimeras mostly devoid of meningeal γδ17 T cells, as well as when meningeal IL-17a was blocked with an antibody administered into the CSF (45). This effect was linked to IL-17a-driven BDNF production by glial cells that enhanced hippocampal neuronal synaptic plasticity to support short-term memory (45). Shortly after, another study demonstrated that the administration of anti-T cell receptor γδ antibodies into the CSF to deplete γδ17 meningeal cells improved performance in the elevated plus maze and open field tests, which was interpreted as reduced anxiety behavior, but did not alter memory function (46). The IL-17a receptor is expressed in cortical glutamatergic neurons, and accordingly its deletion increased anxiety-like behavior in mice (46). Notably, researchers were not able to replicate the previous finding of γδ17 T cell function in short-term memory function, and owing to the limitations in specific ablation of meningeal γδ17 T cells, the exact role of these cells in CNS homeostasis remains unclear. Overall, given the fact that IL-17a has been highly conserved throughout the evolution of the vertebrate immune system and plays key roles in host defense at barrier sites, IL-17a expression by meningeal γδ17 T cells may represent an evolutionary link that serves to protect at barrier sites against pathogens, but also increase the alertness of an animal in new environments conferring survival benefits (46).

While studies have demonstrated a role for meningeal T cell-derived cytokines such as IL-4, IFN-γ, and IL-17 at influencing behavior and cognition, it is currently unclear how these cytokines traverse the meningeal layers and reach the brain. Cytokines in the CSF may reach the brain parenchyma through the glymphatic system along perivascular channels, where they influence neurons through direct receptor signaling (47, 48). Alternatively, in an indirect route, glial cells may first detect the cytokines within the CSF and then release other cytokines, which subsequently impact neuronal activity (2, 42, 48).

### 3.2 B cells

Although B cells comprise a substantial proportion of dural immune cells at steady-state, B cell origin and diversity in the meninges during homeostasis has been explored less extensively than T cells and myeloid cells (7, 37, 49, 50). During steady state in young adult wild-type mice, most meningeal B cells are located within the dura, particularly in the extravascular compartment around blood and lymphatic vessels adjacent to the dural venous sinuses (7, 37, 50). Similarly, within post-mortem human meningeal samples from individuals without inflammatory disease, B cells were often found in the perivascular areas near dural sinuses (7). The dural B cell population in mice is predominantly comprised of the B2 subtype, displaying heterogeneity comparable to that observed in the skull bone marrow but distinct from circulating blood (7, 50). This diverse population included early B cells (IgD^−^CD21^−^CD23^−^IgM^lo^CD24^+^CD43^+^IL7R^+^CD93^+^), immature B cells (IgD^lo^CD22^lo^CD21^lo^CD23^lo^CD24^+^), and mature B cells (MHC-II^+^IgM^+^IgD^+^) (7). This pattern reflected the calvarial origin of meningeal B cells and migration into the dura through osseous channels in the skull bone independent of systemic circulation (7, 37, 50). Many early dural B cells expressed the chemokine receptor *Cxcr4*, and dural fibroblast-like cells had a high expression of its ligand *Cxcl12* (7). Although it remains to be functionally validated, the CXCR4-CXCL12 axis may represent the migratory axis implicated in calvaria-derived B cell homing to the meninges (7). Moreover, dural B cells showed slow turnover and long-term tissue residency, with the dural lymphatic system offering opportunity for exit from the CNS (7, 37, 50). A similar trajectory of developing B cells was identified in a non-human primate, suggesting evolutionary conservation in meningeal B cell development (50). Using a fluorescent reporter system of B cell activation by self-antigens, researchers demonstrated that transitional and mature meningeal B cells have functional B cell receptors (BCRs) (7). Furthermore, transgenic mice with B cells reactive against a myelin oligodendrocyte glycoprotein, a common CNS-epitope, were significantly reduced in the dura compared to other lymphoid and peripheral tissue (7, 50). Collectively, these results suggest that early B cells, supplied by the calvaria, complete their maturation in the dura which provides a local source of CNS epitopes for negative selection. Interestingly, parabiosis studies revealed that unlike early developing B cells, mature dural B cells are capable of exchanging with blood (50). This suggests that the meninges may serve as an additional site of negative selection against peripherally derived B cells that escaped negative selection (**Table 1**) (50).

Meningeal B cells have indirectly been implicated in regulating anxiety-like behavior through myeloid cell activation (51). Chronically stressed mice exhibited a marked decrease in B cells in both the dura and the leptomeninges, but an increase in meningeal myeloid cells (**Table 1**) (51). The transcriptional program of meningeal B cells changed significantly during stressed conditions, with the induction of innate immune transcriptional programs and antimicrobial production genes that likely enable communication with meningeal myeloid cells (51). However, the exact mechanism underlying why meningeal B cells decrease during stress, and their functional impacts on myeloid cell activation within the meninges is yet to be elucidated (51).

During homeostasis, both mouse and human meninges have also been found to contain immunoglobulin-A (IgA^+^) plasma cells, which are terminally differentiated B cells that secrete large amounts of antibodies (5, 37). Sequencing of the B cell receptor (BCR sequencing) revealed that a large portion of these meningeal IgA^+^ cells originated in the intestines. Accordingly, IgA^+^ plasma cells were scarce in the meninges of germ-free mice, but their numbers were restored following gut microbiome re-colonization (5). Interestingly, the composition of the microbiome influenced the repopulation dynamics, with microbiomes derived from two different healthy human donors or a wild-type mouse leading to differences in both plasma cell numbers and the antibody isotype they secreted (5). These results suggest that the composition and variation of the intestinal microbiome can strongly affect the phenotype of meningeal plasma cells during steady-state conditions (5). Given the substantial person-to-person variability in intestinal microbiome profiles, further research in this area will be required to understand how these differences shape the meningeal plasma cell compartment and its functional role in immune surveillance.

### 3.3 Border associated macrophages

The meninges contain distinct populations of tissue-resident macrophages, collectively known as CNS-associated macrophages (CAMs), also referred to as meningeal or border-associated macrophages (52, 53). The distinct characteristics of each meningeal layer create unique niches that shape resident myeloid populations, influencing their transcriptional profiles and creating distinct populations of CAMs within the meninges. Furthermore, regions such as the perivascular space and choroid plexus also contain populations of resident macrophages. The most well-characterized CAMs include leptomeningeal macrophages (mMφ), dural macrophages (dmMφ), perivascular macrophages (pvMφ), and choroid plexus macrophages (cpMφ) (35, 36, 54–58). Additional heterogeneity exists within these populations; for example, cpMφ comprise of distinct stromal cpMφ and epiplexus Kolmer cells (cpepiMφ), while dmMφ and stromal cpMφ CAMs further divide into major histocompatibility complex class II (MHC-II)^low^ and MHC-II^high^ subsets (35, 36, 55).

Tissue-resident macrophages exhibit ontogenetic distinction, originating from either embryonic source, such as the yolk sac and fetal liver, or from circulating bone marrow-derived monocytes during postnatal development (59–65). Fate mapping of embryonically derived macrophages using a tamoxifen-inducible *Cx3cr1* ^CreERT2^ model showed that CAMs originate from erythro-myeloid progenitors (EMPs) of the fetal yolk sac, with no contribution of definitive hematopoiesis during development (56, 57, 66, 67). By embryonic day 10.5 (E10.5), the CNS is colonized by progenitor macrophages that give rise to CAMs and microglia (57, 58). Originally, it was believed that CD206^+^ progenitors gave rise to macrophages and CD206^-^ to microglia, but a recent study has shown that CD206^+^ progenitors can give rise to both cell types (57, 58). Embryonic studies in mice show that by E12.5, meningeal CAMs are present, however pvMφ are only seen at post-natal day 10 (P10), suggesting a postnatal origin for pvMφ. After the development of the perivascular space postnatally, it is seeded by mMφ, which then diverge into pvMφ as the niche matures (57, 58). During adulthood, CAM subsets exhibit heterogeneity in terms of their method of replenishment (35, 56). mMφ, pvMφ, and cp^epi^Mφ exhibit long-term self-renewal with non-significant seeding from blood monocytes (35, 56). On the other hand, dmMφ and stromal cpMφ are largely replenished by blood monocytes (**Table 1**) (49, 51, 56). This suggests that CAMs in permeable brain regions like the dura and choroid plexus stroma undergo replenishment by bone marrow monocytes, while deeper regions rely on long term self-renewal.

Much like microglia, the development of CAMs is dependent on the transcription factors *Pu.1* (SFPI) and *Irf8* but independent of *Myb* (35, 56, 62). Knocking out *Pu.1* leads to a lack of pvMφ, mMφ, and cpMφ, due to a reduction in yolk sac progenitor cells from which CAMs arise. *Irf8* is demonstrated to be a master regulator of CAMs and critical for the divergence of microglia and CAMs (56, 57, 62, 68). In *Irf8* deficient mice, there is a reduction in the number of mMφ at E14.5 (56). Postnatally, *Irf8* is required for expansion of mMφ and pvMφ (58). While the numbers of stromal cpMφ were originally shown to be unaltered in *Irf8*-deficient mice, further studies using macrophage specific *Irf8* deletion showed altered composition of the transcriptional profile of stromal cpMφ without *Irf8*, keeping them in the MHC-II^low^ state and failing to transition into mature MHC-II^high^ cpMφ (35, 56). TGF-βR signaling is dispensable for CAMs but not for microglia development (57, 69). Similarly, transcription factors such as *Batf3* and *Nr4a1* are not required for CAM development (56). As mentioned earlier, pvMφ are derived postnatally at the same time as the perivascular space develops. pvMφ reside within the Virchow–Robin space, where vascular smooth muscle cells (VSMCs) are found (58). VSMCs depend on *Notch3* for development and homeostasis, and in mice lacking *Notch3*, there is a strong reduction in pvMφ. Other CAM subsets are unaffected in *Notch3* knockouts (58). pvMφ also exhibit the need for integrin signaling via *Tln1* and depend on *c-Maf*, a transcriptional factor key for vascular macrophages (58).

CAMs were initially thought to be distinguishable from microglia by their high CD45 expression, whereas microglia are characterized by intermediate CD45 levels (69, 70). However, the discovery of CAM subsets with low CD45 expression in mice highlighted the need for more specific CAM markers (55). Subsequent studies identified CD206, CD36, and Lyve1 as markers specific to CAMs within the CNS (54–56). MHC-II and CD38 are also markers used to identify CAMs while CCR2 helps distinguish dmMφ and stromal cpMφ, as these subsets are replenished by blood monocytes (35, 55, 56). At steady state, CAMs can be identified by a core gene signature consisting of CD206, platelet factor 4 (Pf4), carbonyl reductase 2 (Cbr2), Ms4a7 and Stab1 (36). Additional studies have added Apoe, Ms4a6c, Lyz2 and Tgfbi as universal CAM gene signatures (35). This raises two points of interest: 1) MHC-II^low^ and MHC-II^high^ CAMs are distinct transcriptionally, and 2) cp^epi^Mφ do not exhibit the universal CAM gene signature, rather being more akin to microglial signatures (35). CAMs can also be further distinguished by their lack of α4-integrin and CD44 expression, markers of peripheral monocytes and immune cells respectively (36, 55, 71, 72).

Although significant research has focused on the ontogeny and identity of CAMs, their physiological roles remain poorly understood due to high variability. Positioned at the borders of the CNS, mMφ, dmMφ, and pvMφ contribute to barrier functions and assist in the surveillance and filtering of antigens and metabolic products within the CSF (52, 53). Through receptors like CD206 and the scavenger receptor CD163, pvMφ can uptake exogenous dyes introduced into the CSF, demonstrating their capacity to scavenge and filter this fluid (**Table 1**) (73–75). Recently, CAMs were also shown to be able to uptake peripheral compounds, with MHC-II^+^ CAMs displaying a greater ability to do so (76). In peripheral tissues, vascular macrophages are known to regulate endothelial permeability, suggesting that pvMφ may play a similar role in regulating the BBB (77, 78).

pvMφ also exhibit roles in the regulation of systemic immune responses (79–81). When stimulated by systemic IL-1 and lipopolysaccharide, pvMφ secrete prostaglandin E2, which activates the hypothalamic-pituitary-adrenal axis, ultimately leading to glucocorticoid production and suppression of immune responses (79). The response of pvMφ to systemic IL-1 requires the expression of IL-1 receptors on endothelial cells, indicating a close relationship between these cell types (80, 81). Interestingly, the ablation of CAMs in mice showed that pvMφ can exert direct anti-inflammatory effects on endothelial cells (79).

Using high-fat diet conditions in mice, pvMφ have been shown to induce GLUT1 expression in CNS endothelial cells via the secretion of vascular endothelial growth factor (VEGF) (82). Under similar conditions, pvMφ have been found to contain lipid particles. pvMφ in adipose tissue have been shown to play a crucial role in the regulation of metabolic syndrome, further supporting the notion that pvMφ can play an important role in lipid and glucose metabolism/circulation within the CNS (83).

Future research should focus on CAM ontogeny, plasticity and functionality. This includes investigating the timing, signaling cues, and unique environmental niches that support targeted localization of CAM subsets across meningeal regions. Another important direction is to explore the potential for movement of CAMs between different meningeal regions and if they can assume location specific functions. If CAMs can indeed relocate and take on distinct functions, it would suggest a remarkable functional plasticity and diversity among subsets. Uncovering the precise functional roles of CAMs remains essential, as their contributions to immune surveillance, homeostasis, and responses to injury or disease are not yet fully understood. Research into the transcriptional and molecular diversity within CAM subsets could reveal whether there is redundancy in function or whether these cells are specialized based on their meningeal location, ultimately clarifying the roles CAMs play in maintaining CNS health and responding to pathology.

### 3.4 Dendritic cells

Dendritic cells, characterized as Flt3^+^ and Fcgr1^-^ make up approximately 19% of immune cells found in the healthy dura mater after enzymatic dissociation of the tissue and include conventional type 1 (cDC1) and type 2 (cDC2) subsets, as well as migratory DCs (migDCs), and plasmacytoid DCs (pDCs) expressing Siglech, Ccr9, and Pacsin1 genes (35). Monocyte-dendritic cell progenitors (MDPs) were identified in the skull bone marrow by flow cytometry, indicating local seeding of this antigen-presenting cell (APC) niche by adjacent CNS compartments (**Table 1**) (8). DCs in the meninges are responsible for capturing antigens in proximity to the dural sinuses and presenting them to patrolling T cells (4). Dural sinus-associated conventional type 2 DCs (cDC2s) were found to predominantly take up intra-cisterna magna-administered OVA antigen over the intravenous route, indicating their selective presentation of CNS/CSF-derived antigens (4). Indeed, cDC2s were also found to take up CSF-derived antigens including amyloid-beta (Aβ) peptides, Aβ1-40 and Aβ1-42 (4).

### 3.5 Neutrophils

As for the innate immune system, neutrophils expressing Ly6g, Ngp, Camp, and S100a9 genes, make up approximately 12% of immune cells found in the healthy dura mater after enzymatic tissue dissociation (35). By using spectrally resolved *in vivo* cell labeling in the murine skull, recent literature has evidenced that dural neutrophils are mainly being supplied by the skull bone marrow and not from the periphery after injury (6). Since their discovery in the dura mater and leptomeninges, neutrophils have been identified as key players in brain diseases including ischemic stroke, Alzheimer’s disease (AD) and MS (84, 85). In both the Middle Cerebral Artery Occlusion stroke mouse model and human post-mortem tissue from patients with ischemic stroke, neutrophils were found in the leptomeninges surrounding the infarcted region of the brain (85). Through their production of reactive oxygen species (ROS), proteases, peptidases like MMP-9 that disrupt the BBB, and cytokines that attract other immune cells, these meningeal neutrophils may have a significant influence on the immune landscape in stroke (**Table 1**) (86).

### 3.6 Mast cells

Regarding meningeal mast cells, this population of innate immune cells makes up approximately 2% of immune cells found in the healthy dura mater of mice after enzymatic tissue dissociation (35). Meningeal mast cells have been found to disrupt CNS vascular integrity which is linked to neutrophil recruitment in neuroinflammation (**Table 1**) (87). These cells were further found to influence stroke pathology, as mast cell deficient Kit^W/Wv^ mice had smaller infarcts and reduced brain swelling compared to wild-type controls after stroke (88). However, the role of mast cells in the CNS borders has been more substantially explored in the pathophysiology of migraine. Mast cell degranulation has been found to induce nociception in a calcitonin gene-related peptide (CGRP)-dependent manner (89). Interestingly, there has been recent evidence suggesting that endogenous acetylcholine contributes to migraine pathology mainly through activation of meningeal mast cells (89). Studies in the 5xFAD model of AD pathology have also found routes of communication between the brain and meningeal compartments in the context of mast cells, describing their ability to scan CSF contents for brain antigens and initiate the expression of genes for immune-related effector proteins (90). This may have functional consequences for brain immunity, as an increase in the frequency of disease-associated microglia (DAM) clusters has been described in mast cell-deficient 5xFAD mice (90). However, the exact nature of communication between meningeal mast cells and brain-resident microglia is not yet fully understood.

### 3.7 Innate lymphoid cells and natural killer cells

Innate lymphoid cells (ILCs) and natural killer (NK) cells have also been identified as key players in meningeal immunity (91). From all subsets of ILCs, ILC2s are the predominant subtype in the meninges and localize primarily around the dural venous sinuses (91). Meningeal ILC2s were found to become activated in an IL-33-dependent manner in response to spinal cord injury (SCI) and traumatic brain injury (TBI) (**Table 1**) (91, 92). Nevertheless, their functional implications in disease have not been clearly assessed. However, a beneficial effect on recovery after SCI was indeed observed after addition of wild-type lung-derived ILC2s into the meningeal space (91). In TBI, activation of the metabolic regulator AMP-activated protein kinase (AMPK) led to an increase in IL-13- and IL-5-producing ILC2s, as well as an increase in a distinct IL-10-producing subset of ILC2s in the meninges (92). AMPK-regulated expansion of meningeal ILC2s after TBI was further associated with suppression of pro-inflammatory ILC1 and ILC3 subsets and improvements in motor and psychiatric function in mice (92). ILC2s in the meninges were also found to moderately increase in aging, with a more substantial increase observed in the choroid plexus, and intracerebroventricular transfer of activated ILC2s was associated with enhanced cognitive function of aged mice (93). However, ILC subsets were found to have a contrasting role in the HSV-IL-2 model of MS, as mice lacking ILC2, but not ILC1s or ILC3s, were protected from CNS demyelination (94). This research has come to light despite reports of meningeal ILC1 and ILC3 subsets supporting pro-inflammatory processes in the CNS, indicating the need for closer functional analyses of ILC subsets in the meninges to better understand their complex roles in neuroinflammatory conditions (95–97).

NK cells, a subset of ILC1s, are found to make up approximately 4% of immune cells found in the healthy dura mater after enzymatic tissue dissociation and showed expression of Klrb1c, Ncr1, Eomes and Gzma genes (**Table 1**) (35). Resident NK cells and ILC1s in the meningeal dura have been found to regulate the behavior of adult mice through IFN-γ production (98). Additionally, the production of IFN-γ by meningeal NK cells in a gut microbiome-dependent manner limited CNS inflammation through supporting LAMP1^+^TRAIL^+^ astrocyte induction of T cell apoptosis (99). However, the exact physiological functions of NK cells in the CNS meninges, and how their production of IFN-γ is involved in mediating these functions is poorly understood.

## 4 Spatial organization of immune cells in the dura mater

Although meningeal immune cells are distributed diffusely across the three meningeal layers, several clusters of immune cells have been observed to localize along the dural venous sinuses during steady-state conditions revealing a previously unappreciated level of immune organization in the meninges (4, 5). Comprehensive imaging and single-cell RNA sequencing (RNA-Seq) studies of the dura in naive mice have recently expanded our understanding of the composition and function of these immune clusters, termed the DALT (9) (**Figure 2B**). The largest immune aggregate identified was located in the rostral-rhinal confluence of the sinuses (9). The rostral-rhinal hub contained various immune cells, including CD11c^+^ myeloid cells, T follicular helper cells, T follicular regulatory cells, various B cell subsets (developing, naive, and germinal center), fibroblastic reticular cells, and a complex network of lymphatic vessels and fenestrated vessels (9). Stromal cells within the hub were a source of B and T cell chemoattractant CXCL13. It is plausible other chemokines like CXCL12 are secreted by the stromal cells within DALT, given its key role in lymphocyte migration to the meninges (4, 7). Other cells found in the meninges such as neutrophils, ILC2, monocytes, and macrophages were part of the hub, as well (9) (**Figure 2B**, **Table 1**). Additionally, it was shown that naive B cells could be recruited to the DALT structure from peripheral circulation, but once they were locally activated by T cell interactions within the DALT, a germinal center response could be maintained as evidenced by class-switching and plasma cell differentiation, without further input from circulating immune cells (9) (**Figure 2B**). It is unclear as of yet whether DALT structures can support negative selection of B cells that was previously reported to occur within the dura (7, 50). Similar lymphoid aggregates containing B and T cells have been observed in human dural samples near the base of skull, just above the cribriform plate (9).

The mechanisms governing DALT formation and lymphocyte recirculation within the DALT are yet to be elucidated, as well as the relationship between DALT and responses to aging and different neurological conditions (9). Under homeostatic conditions, these neuroimmune hubs likely serve as regions of immune surveillance. Indeed, with infection and challenge they are seen to grow and respond, as will be discussed later. This lymphoid structure was present in early post-natal animals but expanded in number with age (9). Interestingly, the meninges have been recently identified to play a critical role in promoting CNS tolerance through the expression of CNS-derived autoantigens on APCs, mainly CD11b^+^ CD11c^−^ macrophages (**Table 1**) (18). Particularly, the meninges are enriched in their expression of various myelin-basic protein-derived peptides, including peptides which maintain regulatory populations of T cells in the CNS under homeostatic conditions (**Table 1**) (18). It is unknown if there is a link between DALT structures and tolerance induction in the dura, but plausible as APCs are present within the DALT and dural sinuses are enriched in brain-derived antigen carried in through CSF influx (4, 9).

## 5 Meningeal immunity in disease

### 5.1 Multiple sclerosis

Multiple sclerosis (MS) is a chronic demyelinating CNS disorder that affects over 2.5 million individuals worldwide (100). MS is characterized by the accumulation of myelin autoreactive immune cells and subsequent damage of the myelin, impairing motor and cognitive function. The etiology of the disease remains unknown but is thought to result from a complex interplay of genetic and environmental risk factors (100). Current treatments consist mainly of disease-modifying therapies to control inflammation. Although most of the tissue damage is found within the parenchyma, several studies in humans and animal models have implicated meningeal inflammation playing a critical role in MS pathology (39, 100, 101).

**Figure 3:**
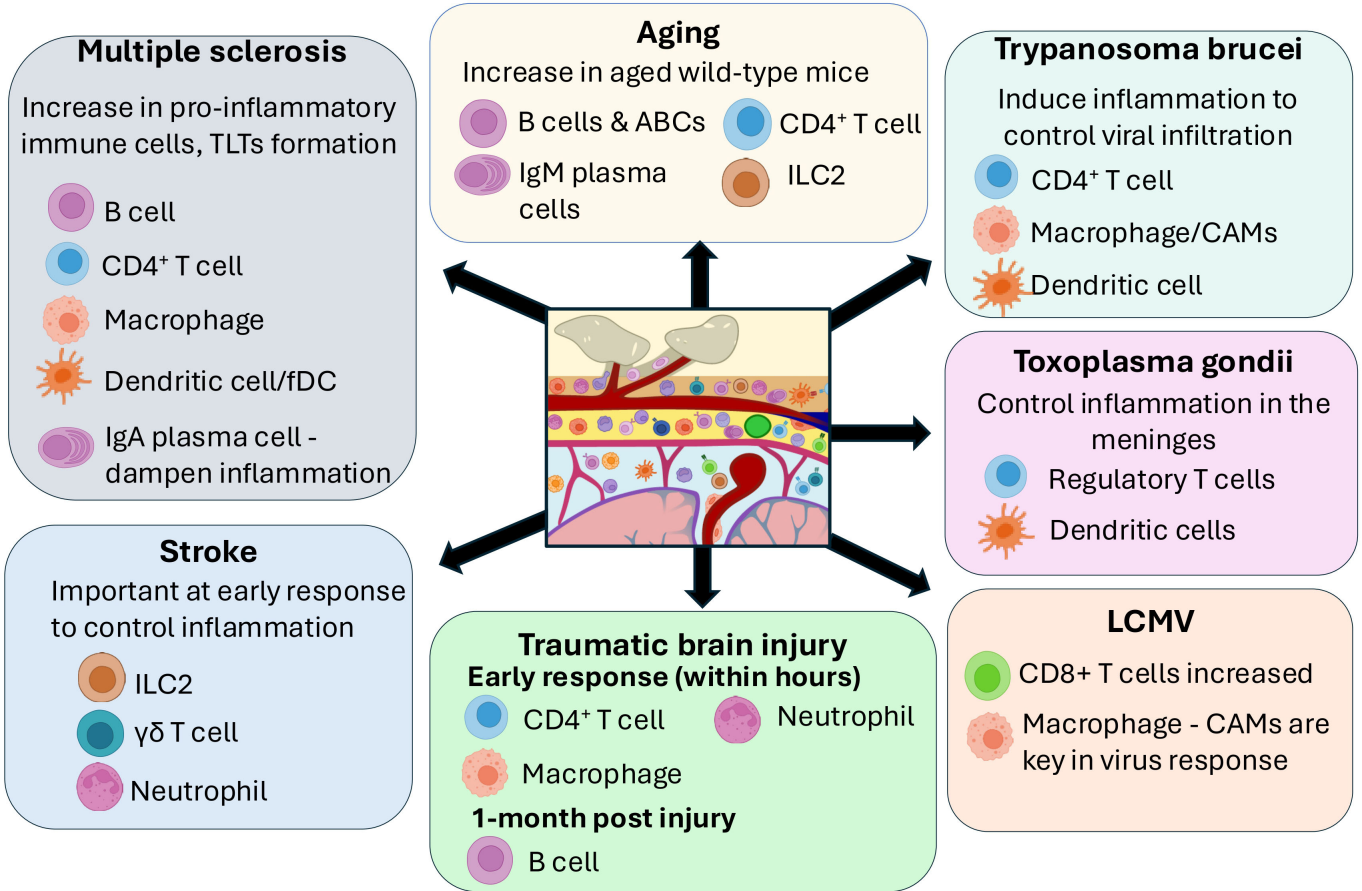
Role of meningeal immune cells in the brain in disease. The importance of different meningeal immune cells subsets in different brain diseases is summarized in this figure. In autoimmune diseases such as Multiple Sclerosis, an increase in proinflammatory immune cells including B cells, T cells, macrophages and DC in the meninges has been described, including formation of Tertiary Lymphoid tissue (TLTs) in the meninges. On the other hand, accumulation of IgA Plasma cells in the dura mater might play a role in dampening inflammation. Stroke and Traumatic brain injury show an important role of innate immune cells such as Type 2 innate lymphoid cells (ILC2) and neutrophils/macrophages in the meninges. Pathogenic brain infections such as Trypanosoma, Toxoplasma and *Lymphocytic choriomeningitis mammarenavirus* (LCMV) have shown an important role of meningeal T cells, dendritic cells and macrophages. On the other hand, aged wild type mice have shown a significant increase of T cells, B cells, Aged-associated B cells (ABCs) and IgM plasma cells in the meninges.

Studies in the widely used experimental autoimmune encephalomyelitis (EAE) animal model of MS-like pathology offer more insight into the role of meningeal inflammation in MS development and progression. EAE can be induced by adoptive transfer of autoreactive T cells (passive EAE) or immunization with myelin antigens (active EAE). After active EAE in SJL mice, meningeal inflammation precedes widespread CNS inflammation, and is characterized by the accumulation of neutrophils, DCs, macrophages, and T cells (102, 103) (**Figure 3**). Similarly, autoreactive T cells are detected in the meninges in early stages of passive EAE (104, 105). Recent findings have highlighted differential contributions of the meningeal layers in cell infiltration during autoimmunity. In both passive and active models of EAE in mice and rats, as well as human post-mortem MS tissue, myeloid and T cell infiltration occurred predominantly in the leptomeninges of the spinal cord and brain compared to the dura (104) (**Figure 3**). The meninges undergo extracellular matrix remodeling and dural and arachnoid fibroblasts show an enrichment of immune functions which promote the migration and retention of immune cells (26). However, transcriptomic analysis suggests that dural vasculature expresses relatively lower levels of adhesion factors which may reduce endothelial cell interactions between effector T cells, as compared to the leptomeninges (104). Furthermore, there is a downregulation of SEMA3A in the leptomeninges, which may further support trafficking of neutrophils, T cells and myeloid cells at ACE points (26) (**Figure 2A**). Imaging of the spinal cord during passive EAE has shown that incoming effector T cells from the periphery crawl and traverse the leptomeningeal vessels in an integrin-dependent manner, appearing in the SAS just one day after adoptive transfer (106–108). The accumulation of encephalitogenic T cells in the leptomeninges is promoted by chemokine signaling through the CXCR3-CCR5 axis and contact with leptomeningeal macrophages through integrin receptors and antigenic stimulation (107). Together, these results demonstrate that the meninges are a critical checkpoint for leukocyte and autoreactive T cell entry into the CNS during neuroinflammation. Effector T cells within the dura and leptomeninges must be reactivated by APCs presenting self-cognate antigen, including macrophages and DCs, before they can invade the CNS parenchyma (36, 37, 109–111) (**Figure 3**). As with cell recruitment, effector T cell reactivation varies across meningeal layers. In a rat model of EAE, leptomeningeal APCs were capable of spontaneously reactivating effector T cells, while dural APCs could present antigen to T cells but required additional autoantigen supplementation to fully stimulate them (104). In a model of passive EAE, single-cell RNA-Seq of myeloid cells identified several neuroinflammation-associated monocyte-derived populations in the leptomeninges. Interestingly, these peripherally derived CD11c^+^ APCs within the leptomeninges (as well as other areas of the CNS) were found to play a larger role in effector T cell activation and disease induction compared to CNS-resident myeloid populations (36). Indeed, high-throughput single cell sequencing on CD45^+^cells isolated from different CNS compartments such as the leptomeninges, parenchyma and choroid plexus have unraveled the CNS myeloid landscape during neuroinflammation (36). Furthermore, there are contrasting results regarding the role of MLVs and draining lymph nodes in effector T cell reactivation during EAE (13, 104, 112, 113). The pharmacological ablation of MLVs and surgical resection of the dCLNs both attenuated EAE severity, likely through limiting antigen sampling and reactivation of T cells (13, 112, 113). Transcriptional profiling of antigen-specific T cells in draining cervical lymph nodes following EAE induction in mice with ablated MLVs compared to EAE mice without lymphatic manipulation showed a reduced inflammatory response of brain-reactive T cells, suggesting that lymphatic drainage in the meninges is critical to fully reactivate antigen-specific T cells in the lymph nodes (13). However, when dorsal and skull base lymphatic vessels were genetically ablated using adenoviral vector expressing vascular endothelial growth factor (VEGF) C/D that blocks essential lymphatic vessel growth factors, no changes in effector T cell infiltration and overall immune cell recruitment to the meninges or EAE severity was observed (104). Overall, the inflamed meningeal milieu in MS supports effector T cell retention and activation that promotes their ability to invade the CNS parenchyma.

The leptomeninges are often sites of tertiary lymphoid tissues (TLT), which are ectopic immune aggregates resembling secondary lymphoid tissues, and are frequently associated with cortical demyelination, axonal atrophy and more severe forms of (39, 114–116). Meningeal TLTs in MS and EAE are B cell-rich, but also contain T cells, plasma cells, DCs and macrophages (102) (**Figure 3**). Infiltrating CD4^+^ T cells expressing IL-17 and Podoplanin were shown to play critical roles in driving the formation of meningeal tertiary lymphoid structures in EAE through stromal cell remodeling to produce extracellular matrix proteins and chemoattractants that promote cell retention (102, 117). In fact, T cells were shown to populate meningeal TLTs first, followed by B cells and other cell types (102). In EAE models, meningeal stromal cells and follicular DCs contribute to the formation of TLT by producing lymphoid-homing chemokines such as CXCL13 and BAFF, which parallels findings in MS patients (102, 118, 119). Infiltrating meningeal neutrophils may also promote the accumulation of B cells in TLTs in an integrin VLA-4-dependent manner (120, 121).

Meningeal TLTs are thought to promote local antigen-specific immune responses that exacerbate chronic disease in MS (122– 124). TLTs have been linked with epitope spreading of myelin-specific T cell responses (122). Additionally, stromal cells that support TLT integrity interact with encephalogenic T cells to produce cytokines that further sustain their responses (102). Several antigen-experienced B cell clones are shared between meningeal TLTs and corresponding brain parenchyma infiltrates in some MS patients (123). The IgH[MOG] transgenic mouse model, which possesses a B cell repertoire biased to recognizing myelin antigen, provides additional insights into the role of B cells within tertiary lymphoid structures in EAE (121, 124). When EAE is actively induced in IgH[MOG] mice, the animals develop severe disease that is characterized by meningeal TLTs (124). The production of IL-23 from meningeal and CNS-infiltrating autoreactive B cells within TLTs promoted inflammation through the maintenance of effector CD4^+^ T cells in the CNS (124).

Meningeal ILCs and mast cells also interact with autoreactive T cells to propagate autoimmunity. Specifically, a subset of T-bet-dependent ILCs localized to the meninges have been shown to support CNS infiltration of myelin-reactive T cells by secreting pro-inflammatory cytokines and matrix metalloproteases (95). Additionally, meningeal mast cells become activated early in EAE and secrete tumor necrosis factor (TNF)-α and IL-1β, aiding in neutrophil recruitment that facilitates BBB dysfunction and inflammatory cytokine production by T cells (87, 105, 125). In mice, the absence of mast cells prevents effector T cell accumulation in the meninges conferring protection from EAE, and reconstitution of meningeal mast cells can restore disease (105, 126). Furthermore, mast cell colocalization with T cells has been observed in post-mortem tissue of some MS patients, reinforcing the idea of interaction between the two subsets (105).Interestingly, meningeal NK cells have been implicated in dampening disease through by IFN-γ production in a gut microbiome-dependent manner. IFN-γ production limited CNS inflammation through supporting astrocyte-mediated effector T cell apoptosis (99).

Overall, a growing body of evidence suggests that meningeal immunity plays a crucial role in the regulation of CNS inflammation and immune surveillance during MS and in animal models of MS pathology. The meninges not only serve as a pathway for autoreactive T cells other effector leukocyte populations to enter the CNS, but also as a route for draining CNS antigens and immune cells. They are also the site of TLT formation which is associated with epitope spreading and sustaining encephalitogenic T cell activity. However, several questions about the immune reactions within the meninges to promote neuroinflammation in EAE and MS remain. While there is a contribution of peripherally derived B cells to the inflamed meninges, recent discoveries demonstrate calvaria-derived B cells develop and undergo negative selection against CNS-derived epitopes locally within the meninges, and B cell-rich DALT structures can sustain local germinal center responses. Further research will be required to clarify the contributions of locally derived B cells compared to peripherally derived B cells in EAE and MS (7, 9, 50, 121, 127). Moreover, a better understanding of the mechanisms guiding tolerance to self-antigens in these spaces under normal conditions, and how this process is disrupted in autoimmune diseases like MS, could offer valuable insights for future therapeutic interventions. Additionally, ectopic tertiary lymphoid structures likely play a key role in supporting immune processes during MS and EAE (39, 102). Understanding processes that govern tertiary lymphoid structure formation better, if they are related to DALT structures present during homeostasis, and how the structures evolve across disease course will be critical to manipulating the balance between anti- and pro-inflammatory reactions within the meninges during neuroinflammation.

### 5.2 Immunity against pathogens

The meninges function as a gateway for neurotropic pathogens targeting the brain parenchyma. CNS interfaces have multiple entry points, enabling immune cells to access the CNS for viral clearance while also serving as sites for pathogen infiltration. This role of the meninges as a critical entry point for pathogens is evident in infections like zika virus (ZIKV) and human immunodeficiency virus (HIV).

Postmortem examinations of neonates with ZIKV showed lymphocytic inflammation and the presence of viral antigens in the meninges (128, 129). Another study found that meningeal inflammation from ZIKV occurred independently of brain parenchymal infection, with meningeal involvement often observed without any corresponding infection in the brain (130). This provided compelling evidence that the meninges serve as a key entry point for ZIKV infection in the CNS. Characterization of the cell infiltrates in the meninges and perivascular space after ZIKV infection revealed elevated levels of macrophages, CD4^+^ and CD8^+^ T cells, and NK cells, along with an increase in cytokine expression, highlighting the meninges as a significant site of immune activation during ZIKV infection (128).

HIV has long been recognized for its ability to infect the CNS and cause neurocognitive disorders, such as HIV-associated dementia. However, the precise mechanisms and timing of its entry into the brain parenchyma remained unclear, with early theories suggesting that HIV infiltrated the brain via monocyte trafficking during the initial stages of infection (131, 132). Post-mortem analysis of human tissues has since shown that the HIV antigen p24 is localized with pvMφ at the CNS interface well before it is detected within the brain parenchyma, suggesting the meningeal immune cells as critical for early viral presence and potential entry into the brain (131–134). In fact, pvMφ have been shown to express CCR5, the necessary co-receptor, required for HIV entry into macrophages (135). Using bioinformatic and phylogenetic analyses to trace viral gene flow, it was determined that HIV not only migrates from the meninges into the CNS parenchyma but also travels from the meninges to peripheral tissues. This bidirectional movement underscores the meninges’ role as both a reservoir and a conduit for viral dissemination within and beyond the CNS (133).

#### 5.2.1 Lymphocytic choriomeningitis virus

Lymphocytic choriomeningitis virus (LCMV) is a pathogen known to cause meningitis in both humans and rodents (136). Previous studies have investigated the immune response to LCMV through intracerebral or intracranial inoculation methods. In these models, LCMV was found to replicate within CAMs and DCs before spreading to peripheral tissues, where it triggers a CD8^+^ T cell response. The resulting pathology and edema in the brain arise when CD8^+^ T cells migrate into the meninges, leading to immune-mediated damage (137) (**Figure 3**). Notably, depleting CD8^+^ T cells before LCMV infection reduces the incidence of CNS disease, yet mice lacking effector pathways commonly employed by antiviral CD8^+^ T cells (such as granzyme, FasL, and IFN-γ) exhibit increased mortality (137). This indicates that CD8^+^ T cells do not induce immune-mediated pathology through direct cytotoxic actions. Instead, it has been demonstrated that the infiltrating antiviral CD8^+^ T cells undergo reactivation in the meninges via both antigen-dependent and independent mechanisms (38), leading to increased release of CCL3, 4 and 5 to attract monocytes and neutrophils from the bloodstream (137). These cells are directly responsible for the vascular pathology associated with LCMV infections. Subsequent studies established that CAMs are infected, activated and killed during LCMV infections, eventually being replaced by blood monocytes. Interestingly, the replacement of blood monocytes decreases the ability for the meningeal immune compartment to responds to secondary microbial challenges, shown to be due to alterations in cholinergic receptor expression (138). Recently, using a peripheral LCMV infection model, it was demonstrated that CAMs play a crucial role in protecting the meninges from LCMV infection, primarily through IFNAR signaling pathways (76).

#### 5.2.2 Trypanosoma brucei

*Trypanosoma brucei* (*T. brucei*), the parasite responsible for human sleeping sickness (African trypanosomiasis) and diseases in other animals, induces inflammation in the choroid plexus and meninges in both rodent and primate models (139–141). Intravital imaging in rodents revealed that following infection, T cells and DCs accumulate in the meninges, with both immune cells and parasites remaining confined to the dura around 40 days post-infection (142). A more recent study demonstrated that *T. brucei* infiltrates the CNS in a stepwise manner–beginning with the meninges, progressing to the choroid plexus, then the CSF, and ultimately reaching the brain parenchyma (143). During this process, monocytes are recruited into the meningeal layers and choroid plexus, while CAMs undergo proliferation. Fate mapping and single-cell RNA-Seq approaches reveal distinct transcriptional signatures between recruited monocytes in the choroid plexus and the cpMφ, suggesting a divergence in the roles of the two cell populations during infection based on ontogeny (**Figure 3**). Depletion of CAMs at early stages of *T. brucei* infection using a Cx3cr1^CreER^:R26-DTR mice (depletion due to diphtheria toxin receptor present on embryonically derived macrophages) led to an increased parasitic load in the meninges (143). However, depletion at later stages of the disease did not result in a similar rise in parasitic burden. This suggests that CAMs play a critical role in the early stages of infection, helping to control the initial parasite load and limiting its entry into the CNS borders. The fact that depletion at later stages did not exacerbate the infection indicates that functional redundancies exist between CAMs and infiltrating myeloid cells, somewhat contradicting the transcriptomic findings. Quite interestingly, as *T. brucei* infection resolves, microglia return to a steady state like transcriptomic signature, whilst CAMs sustain an inflammatory signature and exhibit lasting transcriptomic alterations (143).

#### 5.2.3 Toxoplasma gondii

*Toxoplasma gondii* (*T. gondii*) is a protozoan parasite that can cause chronic CNS infections. T_reg_ cells have been shown to localize to the meninges during toxoplasmic encephalitis in mice and are largely absent from the brain parenchyma (144). These T_reg_ cells utilize the chemokine receptor CXCR3 to remain within the meninges during infection, allowing effector T cells to carry out parasite control within the brain parenchyma (144). A recent study found that during chronic *T. gondii* infection, there was increased activation and expansion of DCs within the dural meninges (145). These DCs were increasingly mature at chronic stages of infection, with the cDC1 subset specifically upregulating markers such as CD80, CD86, and MHC-II. These DCs were also able to capture antigens from the CSF and transport them along local lymphatic vessels to the dCLNs, where they activated both CD4^+^ and CD8^+^ T cells (145). These studies highlight an important role of the meningeal immunity and lymphatic networks at connecting and presenting antigens in the CLN to control of *T. gondii* (**Figure 3**).

#### 5.2.4 Streptococcus pneumoniae

*Streptococcus pneumoniae* (*S. pneumoniae*) is a well-known cause of bacterial meningitis, leading to significant damage through both pathogen-specific virulence factors and host-driven immunopathology. In mice, mMφ and pvMφ have been shown to play a protective role in *S. pneumoniae*-induced meningitis (3). A recent study has also shown an interaction between dural nociceptors and meningeal immunity during *S. pneumoniae*-induced meningitis (146). During infection, *S. pneumoniae* can stimulate these nociceptors, resulting in the release of CGRP within the meninges. CGRP acts to suppress CAM activity, diminishing cytokine production and impairing the recruitment of additional immune cells to the CNS, ultimately exacerbating disease pathology (146). These results not only underscore the complex interactions between the nervous and immune systems in meningitis, but also highlight the need to further explore how neuroimmune signaling modulates immune responses within the meninges during infection.

As discussed previously, the meninges, particularly the dura mater, have been shown to contain B cells and plasma cells, serving as a site for B cell development and selection (5, 9) In mice, the dural sinuses are populated by gut microbiota-dependent IgA^+^ plasma cells. Following intravenous infection with *Candida albicans*, a fungus capable of causing meningoencephalitis in neonates and adults, there is an expansion of B cells and IgA^+^ plasma cells within the dura. These IgA^+^ plasma cells help trap the fungus, and in mice deficient in IgA or lacking meningeal plasma cells, the fungus spreads to the brain parenchyma (5). A more recent study discovered an intricate DALT within the rostral confluence of the sinuses, referred to as the rostral-rhinal venolymphatic hub, and other sites along the dural venous sinuses (9). Intravenously administered antigens accumulated within the DALT resulting in increased numbers of CD45+ and activated B cells within the structure. Furthermore, in response to nasal infection with vesicular stomatitis virus (VSV), the rostral-rhinal venolymphatic hub showed germinal center expansion and somatic hypermutation reactions resulting in the production of antigen-specific B cells and plasma cells (9). The strategic location of DALT along the dural sinuses may help fortify immune surveillance along vulnerable ACE points, where pathogens from the dura may easily access the SAS below. Together, these studies demonstrate the importance of the dural meninges in providing a humoral defense against fungal and viral infections and serving as a key interface between the brain and the body. Future research should investigate how the dynamic variability of the intestinal microbiome shapes humoral immunity in the meninges and influences susceptibility to infectious diseases.

Despite advances in understanding the role of meningeal immune cells, the precise effector mechanisms meningeal immune cells use to fight off pathogens locally remain unclear. There is limited knowledge of whether meningeal immune cells exhibit specialized responses compared to their peripheral counterparts, and little is known about the potential functional heterogeneity of immune cells across the different layers of the meninges in response to pathogens. Future research should focus on uncovering these unique immune dynamics to better understand how the meninges contribute to CNS defense in the context of infection.

## 6 Meningeal immunity in neurodegenerative diseases and brain injury

### 6.1 Stroke

During the acute phase of ischemic stroke (within 24h), innate immune cells are mobilized into the CNS through the production of damage associated molecular patterns (DAMPs), pro-inflammatory cytokines and binding proteins by necrotic cells and activated microglia in the region of the infarcted tissue (147). Inflammatory mediators including TNF, IL-2, CCL2, IL-6, and IL-17 are found to be upregulated in the brain and peripheral blood within hours after ischemia (148, 149). In addition to attracting peripheral immune cells, circulating DAMPs and cytokines cause BBB disruption that allow peripheral immune cells to enter the brain parenchyma and contribute to the inflammatory environment (147). This is followed by significant immunodepression that can leave individuals more susceptible to infectious disease in the days-to-weeks post-stroke (147). In the cervical lymph nodes, into which MLVs drain, higher levels of brain-derived antigens, including MAP2 and myelin basic protein, were found in patients with acute stroke (150). In addition to harboring brain-derived antigens that may be presented to B and T cells, the meningeal lymphatics is an important drainage pathway for CSF that may contribute to the accumulation of neuroinflammatory products in the brain if disrupted after ischemic stroke (151).

Leukocytes in the meninges also respond differently after stroke than those in the brain. A temporary site-specific increase in granulocytes and ILC2s was observed in the dura mater at 24h post-ischemia, as well as a decrease in DCs, NK cells and macrophage subsets and no changes in B cells despite these cells showing an increase in the brain (152). However, neutrophils are more significantly increased in leptomeningeal vessels within hours after experimental stroke (153). CD163^+^ border-associated macrophages participate in granulocyte recruitment, favor the expression of vascular endothelial growth factor and contribute to neurological dysfunction in a focal brain ischemia rat model (154). More recently described IL-17a-secreting γδ17 T cells, which are pro-inflammatory but also implicated in regulating neuronal signaling and cognition in the meninges, are found to be upregulated in the meningeal tissue in ischemic stroke through processes modulated by the intestinal microbiota (149) (**Figure 3**). This sheds light onto some promising research into dietary interventions that may improve post-stroke outcomes by modulation of IL-17a production by γδ17 T cells in the meninges (149). The efficacy of other immunomodulatory therapeutics, including inflammation-mediating minocycline and fingolimod as well as therapeutics that increase T_reg_ expression, are currently being evaluated in clinical trials (155, 156).

### 6.2 Traumatic brain injury

Altered meningeal immunity in TBI is another emerging area of interest that is being explored in human cases and experimental models (157). Immediately post-injury, BBB disruption occurs, and an inflammatory response is initiated through release of DAMPs by damaged neurons and glial cells as well as an influx of neutrophils to the damaged tissue (158). Circulating DAMPs bind to Pattern Recognition Receptors (PRRs) on activated glial cells and macrophages initiating pro-inflammatory cytokines and ROS release, as well as the migration of pro-inflammatory macrophages to the dCLNs to present antigens to naïve T cells and further adaptive responses (159). Traumatic Meningeal Enhancement, associated with meningeal injury and inflammation, has been described in a small sub-cohort of 13% (n=4) of patients with moderate TBI, and 6% (n=2) of patients with severe TBI out of 30 study participants up to 1-year post-injury (160). Studies in mice found severe deficits in the meningeal lymphatic system after TBI that lasted up to 1-month post-injury, and rejuvenation of meningeal lymphatic drainage with viral delivery of VEGF-C was able to ameliorate TBI-induced gliosis (161). In the K14-VEGFR3-Ig model that lack MLVs, a significant reduction in infiltrating CD4^+^ T cells was observed in the brain, suggesting the involvement of MLVs in the neuroimmune response after brain injury (162).

Regarding immune cellular responses in the post-TBI meninges, death of meningeal macrophages was observed within 30 mins of compression injury in mice, followed by infiltration of myelomonocytic cells (monocytes/neutrophils) into the meningeal tissue that scavenge dead cells in a similar manner to neutrophils in the injured brain (47). Pro-inflammatory cytokines IL-1α and IL-1β were found to be elevated in the meninges 6h – 1 day after meningeal compression injury in mice (163). High-throughput RNA-Seq further demonstrated an upregulation of genes related to macrophage and T cell responses at 7 days post-injury, including CD4^+^ Th2 and Th17 cells, CD8^+^ T cells, and NKT cells (164, 165). Increased B cell and IFN gene signatures, including five sub-clusters of B cells, were also observed at 1-month post-injury in the dural meninges of aged mice (164, 165). A neuroprotective effect of meningeal macrophages was also observed through their clearance of extravascular fibrinogen and production of MMP-2 during wound repair post-injury (163) (**Figure 3**).

Given the known role of adaptive immunity in TBI, therapeutics that target inflammatory responses in the injured CNS are currently being investigated. In mice, exogenous administration of IL-33 was found to reduce glial activation and improve the drainage of MLVs to the dCLNs (166). Immunomodulatory therapies have shown efficacy in pre-clinical studies of TBI but failed to improve disease outcomes in larger clinical trials (167). More insight into the contribution of the meninges in seeding immune cells into the brain post-injury, the role of meningeal immunity in long-term recovery, and the effect of aging on this compartment is required to improve the efficacy of immunomodulatory therapies for TBI.

### 6.3 Neurodegenerative diseases and aging

Age-associated changes in meningeal immunity have not been extensively explored in Parkinson’s Disease (PD) and Alzheimer’s Disease (AD). Dynamic contrast-enhanced magnetic resonance imaging of the MLVs in idiopathic PD patients revealed significantly reduced flow of CSF compared to normal controls and patients with atypical Parkinsonian disorders (168). In mice treated with α-synuclein (α-syn) preformed fibrils, blocking MLV flow increased α-synuclein pathology and worsened motor and memory deficits (168). Emergence of α-syn pathology was associated with delayed meningeal lymphatic drainage, significant increases in meningeal macrophages with strong phosphorylated α-syn immunoreactivity, and increased expression of inflammatory cytokines IL-1, IL-6, IFN-γ and TNF-α (168). Singe-cell RNA-Seq and depletion studies in mice further implicated specific subsets of border-associated macrophages (BAMs) in mediating α-syn related neuroinflammation, with these cells playing a role in immune cell recruitment, infiltration and antigen presentation to CD4^+^ T cells (169). BAMs were further identified in human postmortem brain tissues from PD patients in close proximity to T cells (169) (**Figure 3**).

In AD patients and animal models, there has been further evidence implicating the intricate communication networks between innate and adaptive and immune cells in the brain and meningeal layers. Exacerbated glial activation is a hallmark of later stages of AD, as well as vascular damage which together may promote the influx of adaptive and innate cells into the various CNS compartments (170). Impaired meningeal lymphatic drainage was found to be associated with aging, and disruption of MLVs in mouse models of AD promoted the accumulation of amyloid β in the meninges and brain parenchyma (171). Interestingly, near-infrared light was found to modulate lymphatic drainage in mouse models of AD, resulting in improved cognition and alleviation of AD-associated pathology including neuroinflammation (172). A recent study in aged mice demonstrated that IFN-γ production by T cells accumulated in the meninges was a driver of lymphatic impairment, connecting the cellular response to meningeal lymphatic dysfunction seen in aging (173). Evidence for increased numbers of meningeal T and B cells, increased expression of pro-inflammatory cytokines, and increased function such as engulfment in macrophages and other myeloid cells was also uncovered by RNA-Seq in aged mice (174). In particular, a specific population of border-associated macrophages expressing high levels of CD163 and LYVE1 are involved at regulating arterial motion and subsequently regulate CSF flow dynamics, implicated in brain clearance in aging and AD (175). Brain perivascular macrophages located in the perivascular space are involved at increasing oxidative stress and neurovascular dysfunction induced by amyloid beta accumulation in mice (176). Increased expression of border-associated ILC2s was further evidenced in aged mice, mostly accumulated in the choroid plexus, and transfer of *ex-vivo* expanded and activated ILC2s into the brain of aged mice by intracerebroventricular injection was found to alleviated age-associated cognitive decline (93). However, meningeal mast cells were associated with cognitive deficits, and depletion of mast cells in 5xFAD mice elevated the expression of neuroprotective microglia and reduced astrocyte reactivity (90). BAMs in the aged meninges also expressed more AD GWAS genes than other cell types, further highlighting the importance of meningeal immunity in AD development (177).

The dura mater of aged wild type mice harbors a unique population of blood-derived B cells resembling age-associated B cells (ABCs), which have been previously described in the spleen, and had a higher amount of IgM^+^ plasma cells in the dura (7). The amount of IgA^+^ plasma cells do not change when comparing young and old mice (7). The accumulation of ABCs and IgM^+^ plasma cells in the dura with age may endanger the specialized immune environment of the CNS during aging, but their definitive role remains to be defined (7). Furthermore, recent literature has shown how B cells increase in the meninges at early stages of amyloid beta pathology in 5xFAD mice and may exhibit protective effects on AD-like neuropathology by producing the anti-inflammatory cytokine IL-35. However, these cells showed a decline with age (178). In humans, gene microarray analysis of the choroid plexus from postmortem brain tissues of AD patients and normal controls also demonstrated increased expression of EBI3 in AD, a subunit of IL-35, and single-cell RNA-seq data from peripheral blood mononuclear cells further revealed increased variability in B cell expression in human AD as well as the expression of several subtypes of B regulatory cells (178). On the other hand, MHC-II-associated invariant peptide (CLIP)-positive CD19^+^ B cells were expanded in the meninges of aged 5xFAD mice, and antagonizing CLIP was sufficient to reduced CLIP+ B cells and improved both brain pathology and behavioral deficit (179).

In other models of aging, the number of meningeal mucosal-associated invariant T (MAIT) cells increased with age. Single-cell RNA-Seq revealed MAIT cells were enriched in antioxidant defense genes compared to meningeal CD4^+^ and CD8^+^ cells (180). Adult (7-month) MAIT-deficient *Mr1* ^-/-^ mice had decreased expression of tight junction proteins in the leptomeninges and loss of meningeal barrier function that was attributed to an accumulation of ROS in the leptomeninges (180). The loss of barrier function led to microglia activation and hippocampal microgliosis that correlated with impaired spatial learning and memory (180). Interestingly, this phenotype was not observed in young (5-week-old) *Mr1* ^-/-^ mice, highlighting an age-related homeostatic function of MAIT cells, and the existence of potentially compensatory antioxidant systems in younger mice (180).

Despite this evidence of the emerging role of meningeal immunity in aging diseases, many questions remain surrounding the exact mechanisms used by meningeal immune cells to influence processes in the aging brain (**Figure 3**). Elucidating the interplay between reduced meningeal lymphatic drainage and increased activation of meningeal immune cells may help clarify these questions and contribute to the development of new therapeutic targets for age-associated neuroinflammation.

## 7 Discussion and conclusion

Over the last decade, numerous studies have demonstrated that meninges play a critical role in the CNS that goes beyond mechanical protection. The meninges represent a rich immune microenvironment within the CNS that is populated by immune cells derived from embryonic precursors, the adjacent skull bone marrow, and peripheral circulation. The meninges and their immune cells act as a dynamic immunological barrier, shielding the brain from foreign antigens while also mediating tolerance through sampling of brain-derived antigens, and influencing cognition and behavior through the secretion of cytokines. These functional distinctions highlight the unique role of meningeal immunity in maintaining CNS homeostasis and responding to injury or disease, therefore serving as an active immunological site in health and disease.

The recent anatomical discoveries of the glymphatic and meningeal lymphatic systems has provided a clear rationale for the influx and efflux of immune cells within the meningeal layers and the periphery, ultimately draining into the CLNs. A clearer understanding of meningeal anatomy and composition has also shed light onto the complex network involved in the modulation, activation, and migration of immune cells within the skull, meninges and brain. Part of this network also involves complex interactions between resident immune cells and the non-immune cellular components of the meninges, such as stromal cells, fibroblasts, and endothelial cells. For example, the secretion of CXCL13 and CXCL12 by stromal cells in the meninges is crucial to drive the migration and retention of B and T cells within the compartment. Further research is essential to uncover the mechanisms around how non-immune cells contribute to the maintenance and regulation of meningeal immunity and how molecular, metabolic, and genetic cues within the meningeal immune and non-immune cells can define the meningeal immune niche. Additionally, understanding whether certain immune cell interactions within the meningeal niche are redundant, synergistic, or antagonistic could provide insight into the hierarchical structure of meningeal immunity.

As explored in this review, meningeal immune cells dynamically respond to changes, becoming particularly active during disease states such as neuroinflammation, infection, neurodegeneration, and brain injury. There is substantial, yet largely untapped potential in targeting these meningeal immune cells for therapeutic purposes. Therapeutic strategies could involve modulating how these cells respond under pathological conditions or, alternatively, preconditioning them during steady-state conditions to promote a protective response when disease arises.

Furthermore, therapeutic targeting does not have to be limited to the resident immune cells in the meninges. Focusing on the signaling pathways and chemokine gradients within the meningeal environment can potentially influence the recruitment and activity of peripheral immune cells to the meninges. For instance, enhancing chemokine signals to recruit protective immune subsets, while diminishing signals that attract pro-inflammatory cells, could help reshape meningeal immunity to favor neuroprotection and mitigate disease progression.

Despite recent advancements, a deeper understanding of the immune interactions within the meninges is still required. Challenges remain in understanding when and by which mechanisms to target specific immune cells in the meninges either originating from within the CNS compartment, the periphery, or both. This increased understanding will allow for leveraging of the distinctive properties of the meningeal immune niche, offering an opportunity to pioneer novel therapies to protect and restore CNS health across various neurological diseases.
